# Reconstruction-Driven Induction Thermography for AI-Assisted Surface Defect Detection in Welded Structures

**DOI:** 10.3390/s26144422

**Published:** 2026-07-12

**Authors:** Xiang Zhang, Shenghao Huang, Xiaolu Cui, Yu Cao, Dong Wang, Guiyun Tian

**Affiliations:** 1 School of Mechatronics and Vehicle Engineering, Chongqing Jiaotong University, Chongqing 400074, China; huangshenghao@mails.cqjtu.edu.cn (S.H.); cui_xiaolu@foxmail.com (X.C.); caoy16014@gmail.com (Y.C.); 2 State Key Laboratory of Mountain Bridge and Tunnel Engineering, Chongqing Jiaotong University, Chongqing 400074, China; 3 School of Automation Engineering, University of Electronic Science and Technology of China, Chengdu 611731, China; wangdong5240@std.uestc.edu.cn; 4 School of Electrical and Electronic Engineering, Chongqing University of Technology, Chongqing 400054, China; g.y.tian@cqut.edu.cn

**Keywords:** nondestructive inspection, inductive thermography, spatial uniformity, coil occlusion

## Abstract

Welding is widely used to join steel components in energy storage systems, wind power generation systems, and transportation infrastructure. During service, surface defects in steel welds can promote rapid crack propagation and sudden fracture. To address this issue, this study presents an induction thermography framework for post-weld quality inspection of surface-breaking defects in welded steel structures. First, a yoke excitation structure was optimized and evaluated through numerical simulation and experimental validation, and its performance was compared with those of a straight coil and a three-loop coil. The results show that the yoke configuration maintains sufficient heating intensity in the weld region while improving the spatial uniformity of the thermal response. Furthermore, a speed-based reconstruction method was developed to reduce the effects of specimen motion and coil occlusion. By aligning thermal responses from the same physical locations in sequential thermograms, the method reconstructs a continuous temperature field over an extended inspection area. Consequently, the reconstructed images retain clear crack-related thermal features and provide stable inputs for automated analysis. In addition, DeepLabv3+, DSCA-UNet, and feature pyramid network (FPN) were used as representative segmentation models to evaluate the suitability of the reconstructed thermograms for automated defect extraction. On the current laboratory dataset, the three models achieved precision values of 90.4%, 88.6%, and 92.6%, respectively. These results indicate the potential of the proposed framework, while further validation with larger datasets and natural weld defects is still required.

## 1. Introduction

**Figure 1:**
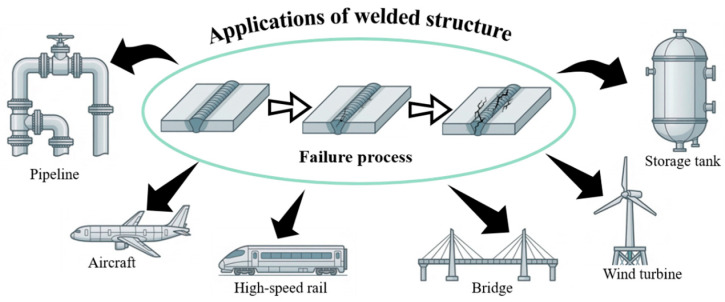
Welded structure in different areas.

Welded structures are widely used in energy storage, wind power generation, and transportation infrastructure, as illustrated in Figure 1. The structural integrity of these structures is essential for the safe operation of critical infrastructure. During long-term service, weld regions are frequently exposed to environmental degradation and cyclic fatigue loading, which can induce different forms of damage. Among these defects, surface cracks are particularly critical because they can reduce the strength, stiffness, and durability of welded components [1,2,3,4]. If these defects are not detected in a timely manner, they may lead to rapid crack propagation and even sudden fracture. Therefore, reliable detection of surface cracks in welds is essential not only for energy security but also for structural condition assessment during service.

Several methods have been used for surface defect detection, including penetrant testing [5,6], magnetic particle testing [7,8], and machine vision inspection [9,10,11]. However, these methods still have limitations when they are applied to surface crack detection in welds. Visual testing depends strongly on operator experience and subjective judgment, which reduces the consistency and reliability of inspection results. Liquid penetrant testing and magnetic particle testing generally require favorable surface conditions and cannot provide intuitive imaging results or sufficient support for in-depth defect analysis. Although machine vision inspection can provide high-resolution images, its performance is highly sensitive to illumination conditions and may be affected by image distortion on complex surface geometries. Therefore, an effective method for detecting surface cracks in welds should provide clear and accurate crack characterization, support non-contact inspection, and incorporate material properties and physical information to enable more comprehensive defect analysis. Compared with the above techniques, thermography has attracted increasing attention in weld surface defect detection.

Among the thermographic approaches, inductive thermography (IT) has become a promising technique for inspecting defects [12]. Previous studies have demonstrated the capability of this technique to reveal surface cracks in metals. Tong et al. [13] investigated the feasibility of IT for characterizing fatigue crack profiles with different depths and validated the method through destructive experiments. Xi et al. [14] developed a multistage collaborative optimization structure and improved the quantitative detection capability of IT for surface cracks in steel plates. Wang et al. [15] combined signal processing and thermal features to achieve effective inspection of complex surfaces using IT. Jiang et al. [16] verified the effectiveness of eddy current thermography for surface defect detection in gears and demonstrated quantitative evaluation of defects with depths of 4 mm. Wang et al. [17] proposed a theoretical model for pulsed thermography with variable pulse width. Despite these advances, two issues remain unresolved in weld inspection. First, the geometric configuration of the excitation coil directly affects the magnetic-field distribution, which can cause non-uniform heating in the weld region and reduce the stability of crack responses. Second, weld reinforcement, coil geometry, and camera viewing angles may obstruct the field of view, thereby limiting the coverage of a single acquisition.

To address these limitations, previous studies have mainly focused on excitation design and inspection strategy. To improve heating uniformity, some researchers have optimized coil geometry or developed dedicated sensing structures to regulate the magnetic-field distribution. These efforts have improved the consistency of thermal excitation across the inspection region. Peng et al. [18] introduced a Helmholtz coil for surface crack detection. This design generated a relatively uniform electromagnetic field in the central region and enabled uniform heating. Tian et al. [19] proposed a U-shaped ferrite sensor for detecting micro-cracks on rail surfaces and compared its performance with that of a conventional excitation coil. Liu et al. [20] proposed an L-shaped inductor, which improved the temperature distribution and expanded the detection area. Xiao et al. [21] developed a dual-coil inductor to enhance temperature uniformity. For field-of-view obstruction, dynamic induction thermography has been proposed as a feasible solution [22,23,24]. Continuous inspection can be achieved through relative motion between the sensing system and the target structure, while line-scan data can be reconstructed to extend the effective inspection range. In [22], the defect on metal surface is realized by moving the specimen at a constant speed. Although this approach improves inspection adaptability, it also introduces new challenges, including substantial data redundancy, difficulty in thermogram alignment, and limited capability for quantitative defect interpretation. Therefore, an efficient processing framework for thermographic data reconstruction and defect feature enhancement is still required. Moreover, the joint optimization of heating uniformity and large-area continuous inspection for weld-specific scenarios remains insufficient and requires further investigation.

In addition, deep-learning-based visual inspection has also been widely investigated for automated crack detection and structural damage assessment [25,26,27,28]. Convolutional neural networks, encoder–decoder segmentation networks, and more recent vision foundation models have been used for crack classification, detection, segmentation, and quantification in concrete, masonry, pavement, and other structural surfaces. These studies demonstrate the potential of deep learning to reduce manual interpretation and improve the automation level of structural inspection. For example, recent studies have reviewed deep-learning methods for structural crack segmentation, detection, and quantification, and have shown that automatic crack analysis has become an important research direction in structural health monitoring. In addition, SAM-based instance segmentation and other advanced deep-learning models have been explored for automated structural damage detection and crack-size estimation.

This study proposes a reconstruction-driven induction thermography framework for AI-assisted surface defect detection in welded structures. The main contribution is the integration of three components into a unified inspection pipeline. First, a weld-specific yoke excitation structure is designed to improve the spatial uniformity of thermal excitation in the weld region. Second, a velocity-compensated thermographic reconstruction method is developed to convert dynamic thermal sequences into spatially consistent reconstructed thermograms while reducing the influence of coil occlusion. Third, representative semantic segmentation networks are introduced to evaluate whether the reconstructed thermograms provide reliable inputs for automated defect extraction. Compared with single-frame thermographic observation or direct image stacking, the proposed framework improves the continuity, interpretability, and AI usability of crack-related thermal features.

## 2. Methodologies

### 2.1. Eddy Current Thermography

Eddy current thermography (ECT) is a coupled electromagnetic (EM)–thermal nondestructive testing technique. During inspection, a high-frequency alternating current is applied to the induction coil, generating a time-varying magnetic field and inducing eddy currents in the conductive specimen. When surface-breaking cracks, micro-cracks, or material discontinuities are present, the local eddy-current path is disturbed. This redistribution of eddy currents modifies the spatial distribution of Joule heating, and the generated heat subsequently diffuses in the specimen. As a result, a defect-related thermal pattern is formed and can be recorded by an infrared camera.

Under the quasi-static assumption, the induced eddy-current field is governed by Maxwell’s equations rather than by an empirical scalar function. The eddy-current field is affected by the excitation current, excitation frequency, coil geometry, lift-off distance, and material properties of the specimen.

Owing to the skin effect, the induced eddy currents are mainly concentrated near the specimen surface and decay with depth. The characteristic penetration depth can be written as

(1) $$\delta =\sqrt{\frac{2}{\omega \mu \sigma }}=\frac{1}{\sqrt{\pi f\mu \sigma }}$$

where δ is the standard penetration depth, ω is the angular frequency, and f is the excitation frequency. Therefore, ECT is particularly sensitive to surface and near-surface defects in conductive materials.

The thermal response is generated by Joule heating associated with the induced eddy currents. For an isotropic conductive material, the volumetric heat source can be written as

(2) $$Q=\frac{|J_{e}{|}^{2}}{\sigma }$$

The transient temperature field is then governed by the heat conduction equation:

(3) $$\rho C_{p}\frac{\partial T}{\partial t}=\nabla \cdot \left(k\nabla T\right)+Q$$

where T is the temperature, ρ is the density, $C_{p}$ is the specific heat capacity, k is the thermal conductivity, and Q is the internal heat generation per unit volume. In this process, heat conduction does not independently identify the defect; rather, it governs the diffusion and spatial spreading of the thermal contrast generated by the disturbed eddy-current field. Therefore, the measured thermal image should be interpreted as the result of coupled EM excitation, Joule heating, and transient heat conduction.

### 2.2. Multiphysics Finite Element Modeling

A 3D finite element model for induction heating is established in COMSOL Multiphysics 6.2. The model consists of the surrounding air domain, the steel specimen, the excitation coil, and the cooling-water domain. The Magnetic Fields interface is coupled with the Heat Transfer in Solids interface, where Joule losses are introduced into the thermal field as a volumetric heat source. A high-frequency alternating current is applied to one end of the coil boundary using the Edge Current feature. The initial ambient temperature is set to 23 °C. The material properties used in the simulation are listed in Table 1.

The magnetic core used in both the simulation and the experiment was Mn–Zn ferrite. In the finite element model, the weld region and the base material were defined as separate material domains. The difference between the weld and the base material was introduced by assigning material parameters to the corresponding regions. The complete welding thermal cycle, metallurgical phase transformation, and residual-stress evolution were not explicitly simulated in this work.

**Figure 2:**
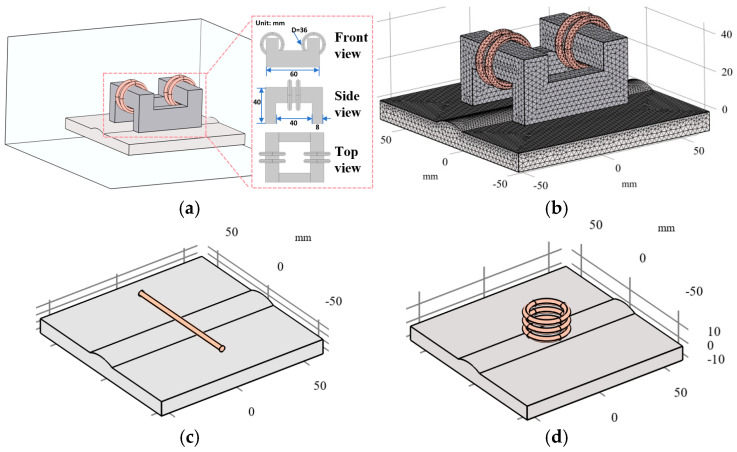
Three-dimensional finite element model for the welded specimen induced by different coils. (**a**) Global and detailed view, (**b**) meshed yoke coil, (**c**) straight coil, (**d**) three-loop coil.

Figure 2a shows the inspection model of the welded specimen excited by the yoke coil. A rectangular air domain of 200 mm × 200 mm × 120 mm is adopted to fully enclose the coil assembly and the specimen. The coil on the yoke was continuously wound and operated in an absolute excitation configuration. The geometrical dimensions of the inductors used in the simulation were kept the same as those used in the experiment. The hollow copper conductor had an outer diameter of 6 mm and an inner diameter of 4 mm. For the three-loop coil, the inner and outer diameters were 45 mm and 57 mm, respectively. For the yoke excitation structure, the magnetic core had dimensions of 60 mm × 56 mm × 40 mm, corresponding to length × width × height. The coil wound on the magnetic yoke had an outer diameter of 36 mm and an inner diameter of 24 mm. The mesh is mainly composed of tetrahedral elements. The element size ranges from 0.5 to 6 mm in the specimen, cooling-water domain, and air domain. Local mesh refinement is applied near the crack to improve the spatial resolution, with an element size ranging from 0.2 to 2 mm in this region. The coil boundary is discretized using triangular surface elements with the predefined fine mesh setting. The resulting mesh is shown in Figure 2b.

Two additional models are constructed to assess the effects of coil geometry on the surface temperature field and heating efficiency. The same excitation current of 200 A was applied in all three simulations. The excitation frequency and heating duration were also kept identical at 100 kHz and 200 ms, respectively. The time step is 50 ms. The straight coil model contains approximately 2.17 × 10^5^ elements, while the three-loop coil model contains approximately 3.26 × 10^5^ elements. All simulations are performed on a workstation equipped with an Intel Core i7-9000 CPU operating at 2.7 GHz and 64 GB of RAM. The yoke coil model requires approximately 25 min to complete, whereas the three-loop coil and straight coil models require approximately 18 min and 12 min, respectively.

### 2.3. Scanning Thermography Processing Workflow

Since the induction coil is located within the field of view of the infrared camera, local temperature information in the weld region is partially occluded, especially for the straight coil configuration. For the three-loop coil and yoke coil, the central inspection region is less affected by direct occlusion, but the single-frame effective inspection area remains limited. As a result, missing data are inevitably present in the acquired thermal sequence. To obtain a spatially consistent temperature field, a motion-compensated line-scanning reconstruction method was applied to the raw thermal data.

In the conventional line-scanning method, a scan line is extracted from a fixed position in each thermogram. These scan lines are then stacked in acquisition order to generate a two-dimensional reconstructed thermal image. This method assumes that a fixed pixel position always corresponds to the same physical location on the specimen surface. However, in scanning induction thermography, relative motion exists between the specimen and the induction coil. Therefore, the image coordinates in the infrared sequence do not fully coincide with the actual coordinates on the weld surface. Direct line stacking would introduce spatial misalignment and reduce the reliability of defect-related thermal response analysis. To avoid this problem, a mapping relationship between the image coordinate system and the specimen coordinate system was first established. The raw thermal sequence was then motion-compensated using the scanning trajectory and velocity information. On this basis, the valid scan lines unaffected by coil occlusion were projected onto the specimen coordinate system. Finally, the temperature data corresponding to the same physical location were resampled and aligned. Through this procedure, a continuous and spatially consistent reconstructed temperature field was obtained, providing a reliable data basis for subsequent defect-related thermal response analysis.

Moreover, temperature contrast ΔT(x,y,t) is used to mitigate the influence of the initial non-uniform temperature distribution on the specimen surface. In this study, t=0 denotes the baseline frame acquired immediately before the excitation current is applied. Therefore, T(x,y,0) represents the initial temperature field before heating, and the temperature contrast is defined as:

(4) ΔT(x,y,t)=T(x,y,t)−T(x,y,0)

where ΔT(x,y,t) is the temperature contrast at time t, T(x,y,t) is the measured temperature field at time t, and T(x,y,0) is the baseline temperature field acquired immediately before heating. This baseline subtraction reduces the influence of initial temperature non-uniformity and highlights the temperature rise induced by electromagnetic excitation.

More specifically, the raw thermographic sequence can be denoted as T(u,v,n), where u and v are the image coordinates and *n* is the frame index. The scanning direction is defined along the u-axis. According to the scanning speed V and the frame rate $f_{r}$, the physical displacement between two adjacent frames is $\Delta X=V/f_{r}$. In this study, V = 20 mm/s and $f_{r}$ = 50 Hz; therefore, ΔX = 0.4 mm/frame. Based on the calibrated pixel size of the infrared image, this displacement is converted into the image displacement in pixel/frame and used to project each valid scan line from the image coordinate system into the specimen coordinate system.

During reconstruction, the purpose of the line-scanning process is not to obtain a single local thermogram, but to recover a continuous thermal image over the scanned weld region. In each raw thermogram, part of the temperature information may be unavailable because the induction coil is located within the camera field of view. Therefore, the region blocked by the induction coil is first excluded from each thermogram, and only the valid scan lines outside the coil-occlusion region are used for coordinate projection. As the specimen moves through the field of view, the same physical location can be observed in different frames when it is not occluded. The valid thermal responses corresponding to the same specimen location are then aligned in the specimen coordinate system according to the scanning speed and frame rate. In this way, a full reconstructed thermogram of the scanned weld region is obtained, rather than a direct stacking result of fixed image lines. The local defect-centered images used for subsequent comparison and segmentation are cropped from this full reconstructed thermogram.

### 2.4. AI-Based Defect Segmentation

Semantic segmentation is a pixel-level image analysis task that assigns each image pixel to a predefined category. From the perspective of digital image processing and computer vision, segmentation transforms a reconstructed thermal image into a binary defect mask, which enables quantitative evaluation of defect morphology, location, and area. In deep-learning-based segmentation, convolutional neural networks extract hierarchical features from the input image, while encoder–decoder structures, multi-scale feature fusion, and contextual modules are commonly used to recover fine spatial details and improve boundary localization [29,30,31,32].

**Figure 3:**
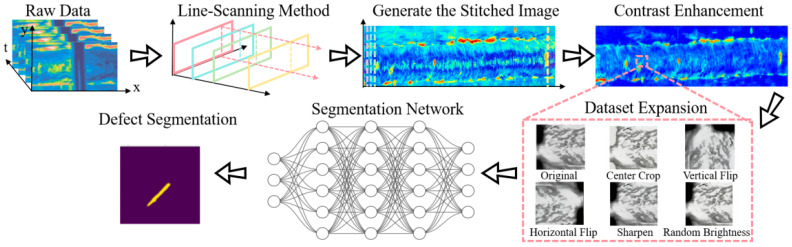
Workflow of experimental data processing.

Three representative semantic segmentation networks, including FPN [33], DeepLabv3+ [34], and DSCA-UNet [35], were employed to evaluate the suitability of the reconstructed thermograms for automated defect extraction. FPN was selected because of its feature-pyramid structure for multi-scale feature fusion. DeepLabv3+ was used to evaluate the effect of multi-scale contextual information on defect segmentation. DSCA-UNet was adopted because its encoder–decoder structure and attention mechanism can enhance defect-related features. These networks were used as representative segmentation models to verify whether the reconstructed thermographic images can provide reliable inputs for AI-assisted weld defect detection. The overall workflow, including thermographic reconstruction, image patch extraction, data augmentation, and defect segmentation, is shown in Figure 3.

After velocity-compensated thermographic reconstruction, AI-based segmentation was performed to automatically extract defect regions from the reconstructed thermal images. In this study, defect detection was formulated as a pixel-level semantic segmentation task. Each reconstructed thermogram was used as the input image, and the corresponding manually annotated defect mask was used as the ground truth. The segmentation model was trained to classify each pixel into either the defect region or the background region.

To prepare the dataset, defect-centered image patches were first extracted from the reconstructed thermograms. This operation reduces the influence of irrelevant background regions and allows the networks to focus on crack-related thermal features. Because the available experimental dataset was relatively small, data augmentation was applied to reduce overfitting and improve the robustness of model training. For each image–mask pair in the training set, the following transformations were used: center crop, vertical flip, horizontal flip, random brightness adjustment, sharpening, and the original image. After augmentation, the total number of training images and their corresponding masks increased to 160.

## 3. Specimens and Experimental Setup

### 3.1. Materials and Specimen Design

**Figure 4:**
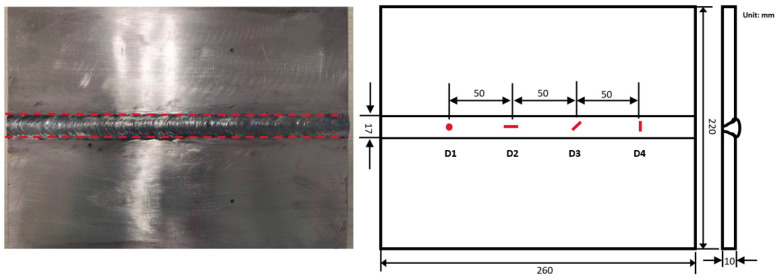
Specimen and defect relative position.

The test specimen was a welded Q345 steel plate with a thickness of 10 mm and a weld width of 17 mm. The specimen was prepared using a V-groove butt-welding process in a flat welding position. To represent typical surface-breaking defects at different locations and orientations in the weld region, four artificial defects were machined by wire electrical discharge machining, as shown in Figure 4. Specifically, D1 denotes a circular defect with a diameter of 3 mm and a depth of 1 mm, while D2, D3, and D4 denote crack-like defects with identical dimensions of 3 mm × 0.2 mm × 1 mm, corresponding to length × width × depth. The crack-like defects D2, D3, and D4 were oriented at 0°, 45°, and 90°, respectively. These artificial defects were introduced to simulate surface-breaking defects that may be detected during post-weld quality inspection after the first welding process. Owing to the small opening width of the crack-like defects, they could not be clearly identified in conventional optical images after machining.

### 3.2. Experimental System

**Figure 5:**
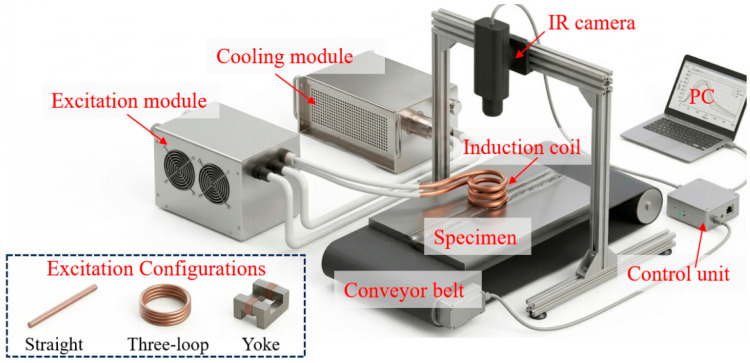
Schematics of the surface detection process.

The experimental system consists of an excitation module, an induction coil, an infrared camera, a central processing unit, and a motion control system, as shown in Figure 5. An Ambrell Easyheat 224 induction heating system was used to drive the hollow copper coil, and cooling water was circulated inside the coil to reduce thermal radiation from the coil itself. The infrared camera was mounted above the inductor and oriented normal to the specimen surface to record the transient thermal response. For the straight coil, three-loop coil, and yoke coil, the relative position between the infrared camera and the inductor was kept fixed, so that the influence of coil geometry on field-of-view occlusion could be compared under the same observation condition. The camera FLIR A655sc has a thermal sensitivity of 20 mK, a frame rate of 50 Hz, and an image resolution of 240 × 320 pixels.

The effective field of view for defect imaging depended on the inductor geometry. For the straight coil, part of the weld region was directly blocked by the conductor, whereas for the three-loop coil and the yoke coil, the central inspection region between the coil or yoke poles was less affected by direct occlusion. Nevertheless, the single-frame inspection area was still limited by the camera field of view and the coil geometry. Therefore, dynamic induction thermography combined with motion-compensated line-scanning reconstruction was adopted to extend the effective imaging area along the weld direction. During inspection, the specimen was moved by a motorized linear stage at a constant speed of 20 mm/s for 16 s. At a frame rate of 50 Hz, 800 thermal frames were acquired, corresponding to a physical displacement of 0.4 mm between two adjacent frames. The acquired thermal sequence covered the defect-containing region and was used as the input for motion-compensated reconstruction. After reconstruction, a full thermographic image of the scanned weld region was obtained, from which local defect-centered patches were cropped for the segmentation dataset.

The heating process in induction thermography was controlled to generate transient thermal contrast rather than sustained material heating. In this study, the heating duration was limited to 200 ms, and no visible discoloration, oxidation, or surface morphology change was observed on the Q345 welded steel specimen after repeated inspections. Therefore, the selected experimental conditions can be regarded as non-destructive for the tested specimen.

## 4. Results and Discussion

### 4.1. Comparison of Different Excitation Configurations

**Figure 6:**
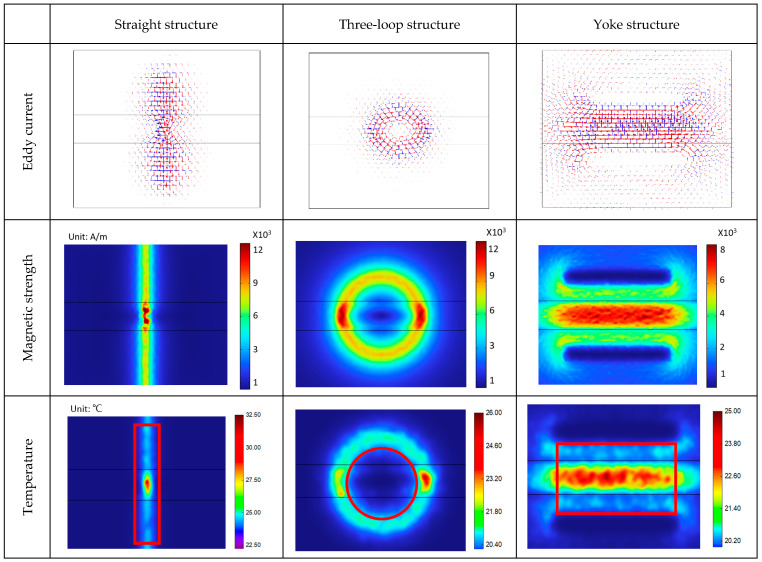
Comparison of different excitation configurations (red box is the region of interest). Blue arrows represent magnetic flux density, while red arrows indicate the current density.

The electromagnetic and thermal responses of different coil configurations are presented in Figure 6. All simulations were conducted under the same excitation conditions: an excitation current of 200 A, an excitation frequency of 100 kHz, and a heating duration of 200 ms. The coil performance was evaluated in terms of heating uniformity, local overheating tendency, normalized heating efficiency, and effective heating area. The uniformity of the heating field was evaluated using the coefficient of variation $CV_{\Delta T}$, where $s_{\Delta T}$ and $\bar{\Delta T}$ are the standard deviation and mean temperature rise in the weld region of interest. A lower $CV_{\Delta T}$ indicates better heating uniformity.

(5) $$CV_{\Delta T}=\frac{s_{\Delta T}}{\bar{\Delta T}}$$

The local overheating tendency was characterized by the peak-to-average ratio defined as:

(6) $$PAR=\frac{\Delta T_{\max}}{\Delta T_{ROI}}$$

The normalized heating efficiency was calculated according to Equation (7), where $P_{in}$ is the input power. In addition, the effective testing area was used to describe the spatial coverage of the heating field.

(7) $$\eta =\frac{{\bar{\Delta T}}_{ROI_{w}}}{P_{in}}$$

The simulation results reveal different heating characteristics for the three excitation configurations in Figure 6, which is used to evaluate the intrinsic heating capability, heating uniformity, and field distribution of different excitation configurations, rather than to demonstrate defect-induced thermal anomalies. The quantitative results of coil performance are shown in Table 2. The straight coil exhibits the highest normalized heating efficiency (η = 4.67), indicating strong electromagnetic coupling and high heating capability. However, this advantage is accompanied by substantial thermal localization, which is not suitable for uniform inspection of an extended weld region.

The three-loop coil provides a broader heating region than the straight coil. Its effective testing area increases to 1590 mm^2^, and both $CV_{\Delta T}$ and PAR decrease to 0.54 and 2.17, respectively. These results indicate that the three-loop coil improves the spatial distribution of the thermal field. However, the enlarged heating area is achieved at the expense of reduced heating strength.

The yoke coil achieves the most balanced performance among the three configurations. It gives the lowest $CV_{\Delta T}$ of 0.40 and the lowest PAR of 1.70, demonstrating the best heating uniformity and the weakest local overheating tendency. More importantly, its effective testing area reaches 2400 mm^2^, which is approximately 2.9 times that of the straight coil and 1.5 times that of the three-loop coil. The yoke acts as a magnetic flux-guiding structure, concentrating and directing the primary magnetic field into the specimen. Consequently, stronger and more spatially distributed magnetic and eddy-current fields are generated in the target region, leading to a broader and more uniform induced thermal field. This characteristic is beneficial for subsequent defect visualization.

### 4.2. Comparison of Numerical and Experimental Methods

**Figure 7:**
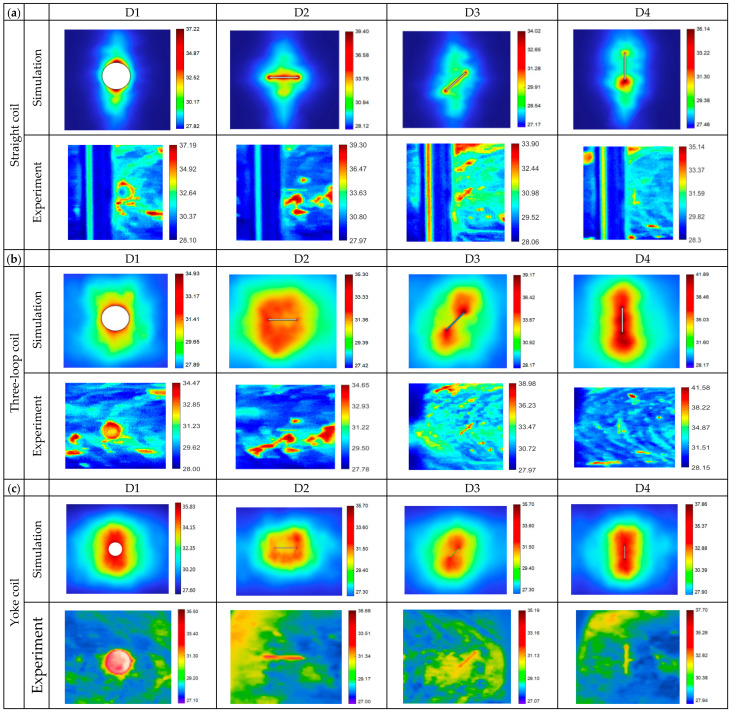
Comparison of tested and simulated results for different coils: (**a**) straight coil; (**b**) three-loop coil; (**c**) yoke coil.

Figure 7 compares the simulated and experimental thermal responses of defects located in the weld-center region under three excitation configurations. Different from Figure 6, which evaluates the background heating field under defect-free conditions, Figure 7 focuses on the defect-induced thermal anomalies after introducing artificial defects into the weld region. In the numerical model, the weld geometry and surface morphology were simplified to focus on the electromagnetic–thermal interaction between the excitation field and the defect. In contrast, the experimental thermograms were affected by the actual weld reinforcement, surface roughness, texture, and local emissivity variations. As a result, the simulated temperature fields are smoother, whereas the experimental results contain stronger background fluctuations. Nevertheless, the simulated and experimental results show similar temperature-distribution tendencies for each excitation configuration.

For defect type, the circular defect is easier to identify than the crack-like defects. Its closed boundary and large effective interruption area disturb the eddy-current field and then produce a compact and localized thermal anomaly. This response is less sensitive to the direction of the induced current and is less likely to be confused with the weld surface texture. In contrast, crack-like defects show clear orientation dependence. This is because the thermal response of a crack is strongly related to its orientation with respect to the dominant eddy-current path. When a crack intersects the induced eddy current more effectively, the current redistribution and Joule-heating perturbation become stronger, producing a clearer thermal anomaly. However, when the crack is closer to the direction of the local eddy-current flow, the current can bypass or redistribute along the crack with a weaker interruption effect, resulting in a narrower and less distinguishable thermal signature. In this study, the 90° crack shows the weakest visibility among the crack-like defects. Its thermal response is further blurred by lateral heat diffusion and is easily mixed with the directional texture and roughness of the weld surface. Therefore, the detectability of crack-like defects is governed not only by defect size but also by the coupling among crack orientation, eddy-current direction, heat diffusion, and weld surface texture.

From the perspective of coil configuration, the straight coil generates the highest temperature rise. However, its heating field is highly directional and non-uniform. The three-loop coil improves the heating uniformity. It reduces the directional bias of the excitation field, but the input energy is distributed over a larger area. The yoke coil provides the most balanced performance. It guides the magnetic flux towards the specimen surface and stabilizes the electromagnetic coupling. This produces a more uniform background and improves the visibility of defect-induced thermal anomalies. The directionality of the excitation field also contributes to the difference among the three configurations. For the straight coil, the induced eddy current is more directional, and the crack response becomes strongly dependent on the angle between the crack and the dominant current path. In contrast, the yoke configuration guides the magnetic flux toward the weld region and provides a more stable coupling condition, which reduces background non-uniformity and improves the visibility of defect-induced thermal anomalies.

### 4.3. Comparison of Different Segmentation Algorithms

FPN, DeepLabv3+, and DSCA-UNet were used to evaluate the suitability of the reconstructed thermograms for defect segmentation. A total of 160 images were used in the experiments and split into training, validation, and testing subsets. Specifically, 120 images were used for training, 20 for model validation, and 20 for testing. All models were trained for 200 epochs with a batch size of 4. The initial learning rate was set to 0.01, and Adam was adopted as the optimizer with a momentum value of 0.9. Bilinear interpolation was enabled during up-sampling. Cross-entropy loss was used as the optimization objective for all semantic segmentation models.

The common evaluation metrics in the field of computer vision include Precision, Accuracy, mIoU, Recall, and F1-score. Precision denotes the proportion of correctly predicted positive pixels among all positive predictions, and Accuracy denotes the proportion of correctly predicted pixels over all predictions. mIoU (Mean Intersection over Union) is the mean value of the IoU (overlap between predicted and ground-truth regions) across all categories. Recall is the proportion of actual positive pixels that are correctly predicted. F1-score is the reconciled average of Precision and Recall, reflecting the overall performance of the network.

(8) $$\mathrm{Precision}=\frac{TP}{TP+FP}$$

(9) $$\mathrm{Accuracy}=\frac{TP+TN}{TP+TN+FP+FN}$$

(10) $$\mathrm{Recall}=\frac{TP}{TP+FN}$$

(11) $$\mathrm{MIoU}=\frac{1}{n+1}\sum_{i=0}^{n}\frac{TP}{FN+FP+TP}$$

(12) $$F1\mathrm{Score}=\frac{2*(\mathrm{Precision}*\mathrm{Recall})}{\left(\mathrm{Precison}+\mathrm{Recall}\right)}$$

where *TP*, *TN*, *FP*, and *FN* denote the number of correctly detected positive class pixels, the number of pixels correctly categorized as background, the number of incorrectly detected pixels, and the number of undetected pixels, respectively.

**Figure 8:**
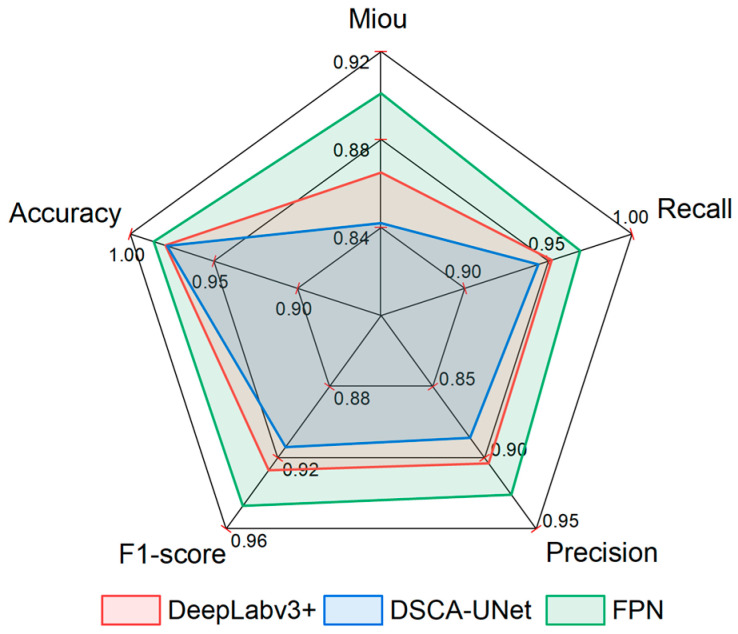
Performance index of the algorithms.

Figure 8 presents the quantitative results for five evaluation metrics. On the current test set, DeepLabv3+, DSCA-UNet, and FPN achieved precision values of 90.4%, 88.6%, and 92.6%, respectively. These results suggest that the reconstructed thermograms contain useful crack-related thermal features for segmentation. However, because the dataset is relatively small and mainly consists of laboratory-fabricated defects, the reported precision values should be interpreted as preliminary evidence rather than a general performance guarantee for all weld inspection conditions.

**Figure 9:**
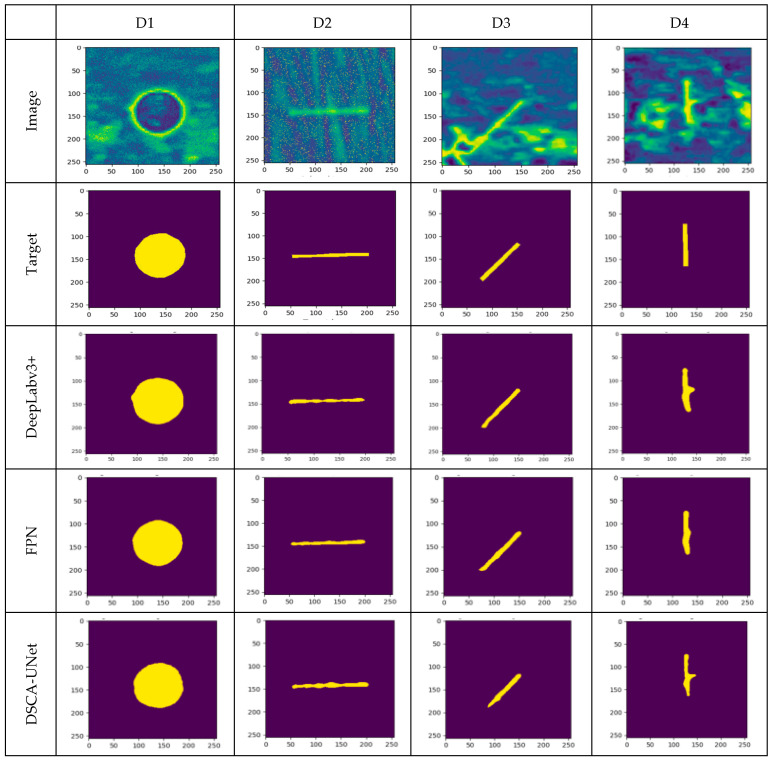
Qualitative results of different defects.

The results in Figure 9 further explain these metric differences. The circular defect is segmented accurately by all models. Its closed geometry produces clear separation from the weld background. For the cracks, the 90° crack shows larger segmentation deviations. The predicted masks exhibit local shape distortion and less accurate boundary delineation near the crack tips. This error is not only caused by the segmentation network but also related to the physical formation of the thermal feature. The 90° crack response is more likely to be coupled with weld texture and directional heat diffusion.

To further analyze the segmentation behavior of different networks, a normalized pixel-level confusion matrix was calculated, as shown in Table 3. Because the number of background pixels was much larger than that of defect pixels, the confusion matrix was normalized within each ground-truth class. Therefore, the TP rate and FN rate were calculated based on the total number of defect pixels, while the TN rate and FP rate were calculated based on the total number of background pixels. This normalization reduces the influence of class imbalance and provides a clearer comparison of missed defect pixels and false alarms.

As shown in Table 3, FPN achieves the highest TP rate of 97.00% and the lowest FN rate of 3.00%, indicating that it has the strongest ability to retain defect pixels among the tested models. Meanwhile, FPN also obtains the lowest FP rate of 0.27% and the highest TN rate of 99.73%, suggesting fewer false alarms in background regions. These results are consistent with the quantitative segmentation metrics and further support the selection of FPN as the most effective model in this study.

The applicability of the proposed method depends on the material properties and defect characteristics. Since induction thermography relies on eddy-current generation and Joule heating, it is mainly suitable for conductive materials, especially ferromagnetic steels. For non-magnetic conductive materials, excitation parameters such as current, frequency, coil geometry, lift-off distance, and heating duration should be further optimized. Crack detectability is also affected by defect size, shape, depth, opening condition, and orientation. Larger or open surface-breaking cracks usually generate clearer thermal anomalies, whereas shallow, narrow, closed, or unfavorably oriented cracks may produce weaker thermal contrast and become more difficult to distinguish from weld texture and background thermal non-uniformity.

## 5. Conclusions

This study proposes a reconstruction-driven induction thermography framework for visualizing surface defects in welded structures. The proposed yoke excitation structure was compared with the straight inductor and the three-loop coil through numerical simulation and experimental validation. A speed-based reconstruction method was also introduced to recover large-field thermal images from dynamic thermal sequences. Several conclusions can be drawn as follows:

(1) The yoke coil achieved a better balance between heating intensity and spatial uniformity in the weld region. Compared with the straight inductor and the three-loop coil, the magnetic yoke guided the magnetic flux toward the specimen surface, improved electromagnetic coupling, and provided a broader and more uniform thermal field for weld inspection.
(2) The proposed line-scanning reconstruction method effectively compensated for specimen motion and coil occlusion. By aligning the thermal responses of the same physical locations, the method reconstructed a continuous temperature field over a larger inspection area.
(3) DeepLabv3+, DSCA-UNet, and FPN were used to evaluate the suitability of the reconstructed thermograms for defect segmentation. On the current laboratory dataset, the reconstructed thermal images provided useful crack-related features for segmentation. However, because the dataset was limited and mainly consisted of artificial defects, further validation with larger datasets and natural defects is required before the segmentation performance can be generalized to practical weld inspection scenarios.

The defects used in this study were artificial defects with predefined sizes and orientations, and the scanning experiment was conducted at a fixed speed of 20 mm/s. Therefore, the reported performance should be interpreted as a laboratory validation of the proposed reconstruction-driven inspection framework rather than a direct guarantee of performance under all practical weld inspection conditions. Future work will further evaluate the method using natural fatigue cracks, different weld geometries, varied surface conditions, and different scanning speeds.

## Figures and Tables

**Table 1:** Material parameters used in numerical models.

Specimen	Relative Permittivity	Conductivity $\left(S/m\right)$	Heat Capacity $\left(J\cdot {\mathrm{kg}}^{-1}\cdot K^{-1}\right)$	Thermal Conductivity $\left(W\cdot m^{-1}\cdot K^{-1}\right)$	Density $\left(\mathrm{kg}\cdot m^{-3}\right)$
Structural steel	200	5.43 × 10^6^	475	52	7850
Mn–Zn ferrite magnetic core	2300	0.15	600	5	7700
Copper	1	5.98 × 10^7^	385	400	8700
Air	1	50	10^3^	2.6 × 10^−2^	1.2

**Table 2:** The evaluation result of the excitation configurations.

Coil Type	$CV_{\Delta T}$	PAR	η	Heating Area (mm^2^)
Straight	0.63	3.56	4.67	840
Three-loop	0.54	2.17	1.08	1590
Yoke	0.40	1.70	1.52	2400

**Table 3:** Normalized pixel-level confusion matrix of different segmentation models.

Model	TP Rate	FN Rate	FP Rate	TN Rate
DeepLabv3+	95.24%	4.76%	0.35%	99.65%
FPN	97.00%	3.00%	0.27%	99.73%
DSCA-UNet	94.49%	5.51%	0.43%	99.57%

## Data Availability

The data presented in this study are available on request from the corresponding author.
